# Supplementary material for: Are carbon emissions trading and green financial instruments synergistic? -Comprehensive quantitative research based on content analysis
Source: PLoS One. 2024 Mar 7;19(3):e0298601. doi: 10.1371/journal.pone.0298601 (PMC10919601; doi:10.1371/journal.pone.0298601)
Supplement: S1 Data — (ZIP) [file pone.0298601.s001.zip › Data/地级市Z_F工作报告.zip!/║■─╧/╓Ω╓▐╩╨/└┤╫╘╣1⁄2╓┌║┼í╛┬φ┐╦╩2╛▌═°í┐_2010.docx]

各位代表：

　　现在，我代表市人民政府向大会作工作报告，请予审议，并请市政协各位委员和其他列席同志提出意见。

　　克难奋进逆势突破提前跨上历史台阶

　　2009年是新世纪以来株洲经济发展最困难的一年。一年来，我们在市委的正确领导下，围绕“保二争一、科学跨越”战略目标，坚持“保增长、强基础、调结构、促民生”和“目标不变、速度不减、标准不降”的方针，超前谋划，沉着应对，提前实现“十一五”规划确定的GDP过1000亿元、财政收入过100亿元、城区面积100平方公里、城区人口100万的“四个一”目标，全市经济社会发展跨越历史性的新台阶。

　　全市GDP达到1020亿元，增长14%；财政收入100.33亿元，增长22.21%，其中一般预算收入66.56亿元，增长42.37%；全社会固定资产投资567.3亿元，增长46.1%；城镇居民人均可支配收入17900元，农民人均纯收入6525元，分别增长12.5%、11.8%；万元GDP能耗下降6%。十大基础工程、十大产业项目和十件民生实事全面完成。

　　（一）保增长，区域经济发展平稳健康

　　工业经济态势良好。实现工业增加值497亿元，增长17%，其中规模工业增加值426.3亿元，增长19.3%。“5115”工程涌现2家销售收入过100亿元、4家过50亿元企业，南车株机公司达130亿元，株冶集团重回100亿元，南车时代、株硬集团、唐人神、中铁轨道过50亿元。启动“6+1+4”产业发展行动计划，轨道交通装备制造、新能源、新材料、医药食品、服饰等产业集群逐步壮大。实施工业投资项目1216个，增加425个，其中投资过10亿元的工业项目8个，过亿元的55个。完成工业技改投资277.9亿元，增长50.5%。园区集聚功能日益增强，新增入园项目79个。

　　农村经济稳步增长。实现农业增加值107亿元，增长5.1%。粮食总量183.9万吨，出栏生猪432万头。农业规模经营再上新台阶，水稻规模种植面积70多万亩，万头以上规模养殖小区35个，年产值过亿元的农产品加工企业15家，农民专业合作组织463家，农业机械化率62%。农村土地流转363万亩，其中耕地流转被誉为“株洲经验”在全省推广。醴陵市获评“全国粮食生产先进县”。“酃县（炎陵）白鹅”获批使用农产品地理标志公共标识，实现零的突破。落实各项支农补贴资金4.96亿元，农民人平政策性增收189元。醴陵、攸县稳居全省县域经济十强，株洲县跻身全省“县域经济发展十快县”行列。

　　第三产业逆势上扬。实现社会消费品零售总额365亿元，增长19.6%。销售家电汽摩下乡产品16.67万台，补贴到位资金6638万元。驰名商标、中国名牌分别达到10件、4个。商品房销售面积425万平方米，增长20.8%。全年接待游客936.46万人次，实现旅游综合收入55.8亿元。进出口总额11.5亿美元。金融支撑作用明显增强，贷款余额485亿元，新增135.7亿元，增长38.8%；新入驻股份制银行2家，筹建小额贷款公司4家。

　　（二）强基础，城乡发展后劲不断增强

　　基础工程强力推进。开工建设一批关系株洲长远发展的重大基础设施项目，全市60项重点工程完成投资129.4亿元。新开工5条高速公路，投资总额160亿元，衡炎高速建成通车，实现“县县通高速”。衡茶吉铁路、长株高速、炎睦高速有序推进。武广高铁株洲西站投入运营，争取沪昆高速株洲东设站。干线公路改建提速，省道S313、S320、S321公路完成改建，网朱公路攸县段、衡炎高速安攸互通连接线、国道106炎帝陵牌坊至炎陵县城公路等基本建成，醴潭高速芷线桥至渌口公路完成路基建设。

　　城市品位明显提升。城市提质扩容步伐加快，王家坪立交、炎帝大道、时代大道建成通车，红旗路改造如期完成，田心立交基本建成，迎宾大道、株洲大道延伸段、湘江风光带建设顺利推进。创卫工作卓有成效，荣获“国家卫生城市”称号；国家交通管理模范城市、国家环保模范城市、全国文明城市创建工作深入推进，大部分指标达到国家考评标准。“绿化、美化、亮化、数字化”顺利完成，新增绿地51.5公顷，城市绿化覆盖率提高到43.6%；美化临街建筑物1511栋，拆除防盗窗4.2万个；亮化临街建筑282栋，新建33条小街小巷路灯照明；数字化城市管理系统建成运行，城市治安电子防控系统、智能交通管理系统不断完善。城市更加清新、更具品位、更富现代气息。

　　农村基础逐步夯实。建设新农村综合示范片22个，扶贫开发示范村10个，云田村、仙庾村被列为全省新农村建设重点示范村。改造通乡公路153.9公里，建成通畅工程603公里、农村客运站12个、农村客运招呼站206个，新建沼气池10364口，新解决13.56万农村人口饮水不安全问题。完成各类水利工程2万处，治理病险水库97座。洮水水库主体工程基本建成，桐坝水电站开工建设。林权制度改革全面实施，改革完成率达60.6%，森林覆盖率提高到59.79%。茶陵县获评省级卫生城市，炎陵县入选第二届“中国十佳绿色城市”，云阳山自然保护区被列为省级自然保护区、省级风景名胜区和省级地质公园。

　　（三）抓改革，“两型”社会建设实质推进

　　“两型”规划基本完成。《株洲市“两型”社会建设综合配套改革总体实施方案》、《株洲市核心区发展战略规划》等6个总体性规划和方案通过评审，《株洲市“两型”社会建设产业发展规划》、《株洲市土地利用总体规划》等7个专项规划全面完成。

　　示范区建设全面铺开。云龙示范区高水准的规划顶层设计基本完成，组建云龙示范区工作机构和云龙发展投资控股集团有限公司，争取长期战略合作协议资金111亿元。云峰大道、长株高速云田互通开工建设。投资150亿元的湖南华强文化科技产业基地和投资过100亿元的职教城等项目启动建设。清水塘循环经济工业区45个项目列入长江中下游流域水污染防治规划和国家循环经济试点项目库，12个节能减排项目和5个限期治理项目相继实施，铜霞路等道路建设顺利推进。天易示范区北汽控股南方生产基地、风电产业园等项目相继开工建设。

　　综合改革有序推进。制订《株洲市统筹城乡发展改革试点总体方案》和7个专项方案，白井片区成为首个省级统筹城乡发展改革试点区，云龙示范区云田村、株洲县松西子社区等地的试点顺利展开。城乡建设用地增减挂钩、土地征转分离改革稳步推进，天元区高塘村率先试点成功。成品油税费改革领先全省。投融资体制改革全面铺开，按市场理念重组城发集团、国投集团、地产集团，发展能力明显增强。

　　环境保护得到加强。化学需氧量、二氧化硫排放量得到大幅削减，提前完成“十一五”砷、镉减排任务。列入省、市政府环境污染限期治理的16家企业全部达标。清水塘地区重点水污染治理项目和湘江污水治理项目取得阶段性成果，霞湾港水变清一期工程全面完成，湘江株洲段水体质量保持国家Ⅲ类标准。市区饮用水源安全得到保障。建成城镇污水处理厂7个，提前一年实现城镇污水处理设施建设三年行动计划目标。成功获批全国可再生能源建筑应用示范城市。

　　（四）调结构，经济发展格局更趋合理

　　三次产业协调发展。三次产业比由上年的12.1：54.6：33.3调整为10.6：55.4：34，结构不断优化。规模工业总产值过千亿元，规模工业新产品产值占工业产值的18%，提高2.6个百分点。高新技术产品增加值占工业增加值的40.6%，提高3个百分点。

　　非公经济不断壮大。非公有制经济实现增加值570亿元，增长15.5%，占全市GDP总量的55.9%。新增产值过亿元的非公企业25家。非公经济完成城镇固定资产投资366.9亿元，增长55.6%。非公规模工业实现增加值230亿元，增长26.5%。

　　投资结构逐步优化。农业、工业和基础设施投资稳步提高，分别增长24%、50%、40%；技改投资和新兴产业投资占工业投资的89.2%。招商引资卓有成效，引进内资项目178个，实际利用内资94.82亿元，增长29.35%；新批外商项目61家，实际利用外资3.62亿美元，增长20.53%；荣获“中国最佳粤商投资城市”称号。

　　（五）促民生，人民群众生活继续改善

　　社会事业全面发展。教育事业蓬勃发展，高中学业水平考试总合格率全省第一。建设合格学校53所，新建塑胶运动场12个，化解义务教育债务1.5亿元，资助贫困学生4.82万人次。职教城成为推动湖南新型工业化进程的重大项目，湖南铁路科技职业技术学院、湖南有色金属职业技术学院、湖南工贸技师学院新校区开工建设。科技事业成绩突出，第七次荣获全国科技进步先进城市，获评国家知识产权工作示范城市；获批上级各类科技项目80个；成功研制出我国首列高端A型地铁车辆，引进IGBT生产核心技术，填补两项国内空白；在全国率先推行城区公交电动化三年行动计划，180台混合动力公交车投入运行。文化事业亮点纷呈，文化惠民工程顺利推进；成功承办2009年湖南省艺术节，荣获“全国全民阅读活动先进集体”称号。广播电视中心基本建成。体育事业加快发展，八一男排落户株洲，市十一运会成功举办，全民健身活动深入开展。医药卫生体制改革稳妥推进，市中心医院主体工程提前完工，新型农村合作医疗参合率92.57%，平均住院费用报销率42.27%，甲型H1N1流感等重大疾病得到有效防控。人口计生工作成效显著，符合政策生育率91%，连续19年被评为“全省人口计生工作先进”。社区居委会换届选举圆满完成，直选率56%。民族宗教工作扎实有效，连续五次获得“全国民族团结进步模范集体”称号。妇女儿童、气象、水文、市志、档案、人防、扶贫、助残、老龄等工作取得新成效，国防动员、民兵预备役工作扎实推进。

　　保障水平不断提高。新增城镇就业4.41万人，稳定农村劳动力转移就业69.26万人，援助农村贫困家庭转移就业1.2万户。政府投资2000万元开发公益性岗位，持续保持城镇“零就业家庭”动态为零，城镇登记失业率控制在4.5%以内。社会保险覆盖面不断扩大，企业养老保险新增参保6.94万人，城镇居民医疗保险参保人数增至71.19万人。新型农村养老保险在株洲县展开试点。城乡低保水平进一步提高，月人均补差分别达到170元、53元。新建改造农贸市场21个。新建农村安居房1085栋，廉租住房5917套，首个廉租住房示范项目“和谐家园”一期投入使用。改扩建敬老院7所、五保之家19所，五保对象集中供养率提高到25%。

　　社会大局保持稳定。切实加强信访工作，信访总量大幅下降。大力加强综合治理基层基础工作，社会服务和管理机制逐步完善，打击和预防犯罪机制不断健全，各种刑事犯罪活动逐步减少。公众安全感和满意度明显增强，连续两次获得社会治安综合治理“长安杯”。不断强化生产安全、交通安全、消防安全、食品药品安全和质量技术监管，应对突发事件能力不断提高，人民群众生命财产安全得到有效维护。

　　与此同时，政府自身建设同步加强。自觉接受人大的法律监督和政协的民主监督，认真听取民主党派、工商联和无党派人士的意见，办结人大代表建议283件，政协委员提案406件，办结率、见面率、满意率均为100%。行政许可网上审批及电子监察系统投入运行。推行项目审批代理制、限时办结制、项目代建制等行政管理体制改革，减少行政许可项目68项。实行科学民主的决策机制、精简效能的执行机制、奖罚分明的激励机制、权责统一的问责机制，行政效能得到提高。“12345”市长热线开通运行。深入推进“四治”活动，公务员作风建设明显加强。强化行政监察、审计监督，廉政建设和反腐败工作扎实有效。

　　各位代表！2009年，是株洲发展极不平凡、极其艰难、极富成效的一年。这一年，我们成功抵御金融危机的强烈冲击，经济低开高走，取得超出预期的业绩；这一年，我们成功经受建设高峰期和偿债高峰期的严峻考验，城市和交通基础设施建设成效显著，城乡面貌极大改观；这一年，我们提前完成“十一五”规划确定的“四个一”目标，实现了株洲几代人的梦想，株洲由此进入了一个加快发展的新阶段。我们深切体会到，做好政府工作，必须以人为本，关注民生；必须激情工作，规范管理；必须勤政廉政，依法行政。我们深切体会到，要保持良好发展态势，就要坚持“一化三基”发展战略不动摇；

　　就要坚持“保二争一、科学跨越”战略目标不动摇；就要坚持“以工业文明为特征的生态宜居城市”发展定位不动摇。我们深切体会到，成绩的取得，是市委正确领导、市人大和市政协支持、监督的结果，是各民主党派、工商联、无党派人士、各人民团体参政议政的结果，饱含了各位代表和委员的智慧心血，凝聚了全市人民的辛勤汗水。在此，我代表市人民政府，向各位人大代表和政协委员，向驻株部队官兵和全市人民，向关心、支持、参与株洲建设的各界人士，致以崇高的敬意和诚挚的谢意！

　　在总结成绩的同时，我们也清醒地认识到发展过程中还存在一些问题和不足，主要是：经济发展方式仍需转变，经济增长基础仍不稳固；产业结构仍需调整，部分产业和企业抗风险能力较弱；投融资主体市场运作欠缺，中小企业融资仍然困难；城乡就业形势严峻，农民增收途径有限；安全生产仍存有隐患，社会稳定压力较大；行政执行力有待提升，政务环境仍需优化，等等。我们将高度警醒，认真对待，切实解决。

　　审时度势准确把握科学谋划全年工作

　　2010年，全球经济复苏缓慢曲折，国内宏观政策稳中趋紧，将加大株洲发展的难度。但国家中部崛起战略的实施，“两型社会”建设的推动和株洲“保二争一”的来势，表明株洲经济社会加速发展的态势已经形成。我们将按照市委“目标提高、标准提升、发展提速”的总体要求，切实转变发展方式，实现株洲经济社会超常规发展。

　　2010年政府工作的指导思想是：坚持以邓小平理论和“三个代表”重要思想为指导，深入贯彻落实科学发展观，围绕“两型”社会建设，解放思想，开拓创新，转方式、调结构、强基础、惠民生，突出重大项目建设、重点地区发展和重要领域改革，加快推进新型工业化、新型城市化和新农村建设，推动经济社会又好又快发展，夺取“保二争一、科学跨越”的新胜利。

　　全年经济社会发展的目标是：GDP增长13%以上；财政收入、一般预算收入均增长13%以上；全社会固定资产投资增长35%以上；社会消费品零售总额增长18%以上；城镇居民人均可支配收入增长12%以上、农民人均纯收入增长10%以上；人口自然增长率控制在8.4‰以内；万元GDP能耗和主要污染物排放总量均下降5%以上；各项事业都有新的进步。同时，集中人力、财力和物力，重点打好“三大战役”，突出抓好十大基础工程、十大产业项目和十件民生实事。

　　三大战役：1.城市提质。就是要深化提质的内涵，建立健全城市管理长效机制；扩大提质的外延，实现城市提质向“全域株洲”延伸；提升提质的水平，实现城市的发展定位。2.园区攻坚。要在提升园区影响力上攻坚，打造示范区，建设新株洲；要在放大园区贡献力上攻坚，形成项目洼地，推动百亿、千亿园区建设；要在增强园区带动力上攻坚，加速产业集群，实现集约发展。3.旅游升温。要推动旅游品牌升温，走精品发展的路子；要推动旅游规模升温，走科学开发的路子；要推动旅游市场升温，走现代营销的路子，努力把旅游业培育成株洲新的支柱产业。

　　十大基础工程：1.建成长株高速，加快醴炎、炎睦、泉南高速茶陵段等六条高速公路建设，完善区域交通网。2.建成迎宾大道、株洲大道延伸段，完善城市“四张大门”。3.建成湘江五桥，形成城市内环。4.建成湘江风光带河西城区段，提升城市品位。5.力争建成市中心医院并投入使用，改善就医条件。6.打通长江路、铜霞路、株醴路、石宋路，启动云龙大道、荷塘大道、航空大道建设，完善城市路网。7.建设垃圾焚烧发电厂和重金属工业废水处理厂，提升垃圾无害化和污水处理水平。8.加快建设神农城，打造城市名片。9.加快铜塘湾港区和物流园区建设，提升物流优势。10.加快职教城建设步伐，打造“中部地区职业教育创新之都”。

　　十大产业项目：1.建设千亿产业园区，打造轨道交通千亿产业集群。2.实现南车时代百亿工程，打造行业旗舰。3.完成北汽控股南方生产基地建设，实现轿车下线。4.推进湖南华强科技文化产业基地建设，力争一期工程开园面世。5.加快攸县煤电一体化项目建设，力争完成攸县电厂项目主体工程。6.加大有色产业技改力度，形成精深加工基地。7.加快航空城建设，发展民用航空高技术产业。8.基本建成风电产业园，培育战略性新兴产业。9.加速釉下五彩陶瓷创意产业园建设，打造百亿产业园区。10.加快建设神农福地和湘水湾生态公园，打造文化娱乐休闲胜地。

　　十件民生实事：1.积极扩大就业。新增城镇就业4.41万人、失业人员再就业2.2万人、“零就业家庭”动态援助100％、新增农村劳动力转移就业6.6万人。2.破解就医难题。新型农村合作医疗参合率巩固在90%以上，城镇居民医疗保险住院补偿率稳定在42%以上；启动城市四区、株洲县基本药物零差价销售。3.改善就学条件。建成义务教育合格学校20所；资助家庭贫困学生1.65万人。4.加强住房保障。新增廉租住房6000套；新建农村安居房1000栋；新增廉租住房租赁补贴家庭1000户。5.完善社保体系。启动城市四区和云龙示范区新型农村养老保险，新增参保人数26万人（含株洲县）；新增企业养老保险参保人数6.5万人，稳定城镇居民医疗保险参保人数68万人；城区低保保障线标准提高到300元，农村低保相应提标。6.保护生态环境。加大“一江四港”治理力度，确保湘江株洲段保持国家Ⅲ类以上水质；城市污水处理率、垃圾无害化处理率分别达到80%、100%；新增混合动力公交车260台；建设清洁工程示范村8个。7.新建改造农贸市场。城区新建改造农贸市场20个，每个县市至少完成1个示范点。8.创建平安株洲。重点单位、要害部位技防设施安装联网率达100%。9.加强社会救助。基本完成市儿童社会福利院和市救助管理站迁建工程；新建和改扩建敬老院6所，五保对象集中供养率提高到28%。10.保障食品安全。健全食品药品质量安全监管体系，确保蔬菜农药残留超标率控制在8%以内，生猪“瘦肉精”检出率低于1%；基本药物目录考核品种质量合格率98%以上；新解决13万农村人口饮水不安全问题。

　　围绕上述指导思想和发展目标，做好今年政府工作，我们务必坚持科学发展这一主线。更加注重资源节约、环境友好，更加注重提高经济增长质量和效益，更加注重推动经济发展方式转变和经济结构调整，增强经济整体素质和发展后劲。我们务必把握改革创新这一关键。更加注重用国际视野和世界眼光认识自我，把握形势，切实解放思想，更加注重推进改革开放和自主创新，深化关键领域改革，增强经济增长活力。我们务必筑牢市场运作这一理念。坚定不移地运用市场手段整合各类资源，促进政府“有形的手”与市场“无形的手”良性互动，努力实现资源资产化、资产资本化、资本证券化。我们务必遵循城乡统筹这一途径。坚定不移地构建平等统一的城乡制度框架与政策体系，加大农村投入，统筹城乡产业发展、基础建设和公共服务，促进城乡相互融合、互动发展。我们务必抓住改善民生这一根本。坚定不移地深化惠民行动，更加注重改善民生，保持社会和谐稳定，切实做到发展尊重人民、发展依靠人民、发展为了人民、发展造福人民。

　　突出重点乘势而上致力推动科学跨越

　　（一）突出“两型”建设，推进综合配套改革

　　推动“两型”改革率先突破。发挥“两型”改革试验区先行先试的权利，全面推进各项改革。重点推动城乡统筹改革，促进基础设施一体化、公共服务均等化、社会保障同城化、社会管理社区化、土地利用集约化、产业发展集聚化，积极探索解决城乡二元结构所带来的社会问题，统筹城乡协调发展。推进土地经营制度改革，统筹全市土地储备和经营，调控土地供应闸门，探索建立农村宅基地和农村集体建设用地地票交易制度，为加快城市化步伐和农民增收拓展新的空间。深化投融资体制改革，整合政府资源，按照资源资产化、资产资本化、资本证券化的市场运营理念，打造推动城市和园区建设以及产业发展的航母，使之成为市场主体，防范财政风险。启动城区建设体制改革，适度下放城区建设权，理顺责权利关系，形成多元投入、两级分担、社会共建、全民参与的城市建设格局。

　　推动示范区建设率先作为。云龙示范区要按照“生态城、文化城、科教城、旅游城”的定位，加强基础设施建设，加快云峰湖国际度假区和职教城的建设步伐，早日形成示范效果。清水塘循环经济工业区要迅速启动轨道交通千亿产业园、国际环保工业园、物流园的建设，加大铜塘湾港区、工业污水处理厂等基础设施建设力度，显现循环经济效应，让老工业区焕发新的生机。天易示范区要发挥国家高新区的聚焦、辐射和带动作用，加快构建“两型”产业体系，发展电动汽车、风电装备等战略性新兴产业，尤其要加快栗雨工业园中央商务区、湘水湾生态公园、神农城、武广新城和新马片区的建设，打造城市精品。

　　推动环境治理率先见效。以创建国家环保模范城市为契机，加大重点工业污染源治理，加快淘汰落后产能，加强环境保护能力建设。抓住湘江列入国家重点流域治理的重大机遇，加强“一江四港”综合治理。推进清水塘地区环境污染综合整治，加快34.41平方公里重金属污染土壤的生态修复和综合利用，切实改变清水塘地区的面貌。推进城镇、农村治污工作，加强农村面源污染治理。

　　（二）突出结构调整，提高经济发展质量

　　帮扶企业发展。推进“5115”工程，支持南车株机公司、南车时代、中铁轨道等企业做大做强，打造轨道交通装备制造千亿产业集群；支持北汽控股迅速达产，规划汽车千亿产业集群。支持株冶集团、株硬集团、千金药业、智成化工等企业加快裂变，引导企业产品终端化、经营多元化、销售规模化、产业园区化，提升企业核心竞争力。扶持中小企业发展，推进企业技术改造、自主创新和兼并重组，将中小企业纳入产业集群内分工协作体系。启动总部经济基地建设，逐步形成梯次跟进、大小并举的企业发展格局。鼓励企业进入资本市场，指导旗滨集团、天桥起重、唐人神等一批企业上市。构建中小企业信息和决策服务平台，做好企业市场引导和信息服务。促进政银企交流对接，搭建中小企业担保、同行业互保、信贷风险补偿、信贷应急平台，营造诚信金融环境，最大限度发挥金融对经济的支持作用。

　　调整产业结构。调整工业结构。坚持“定好一个产业、做好一个规划、组建一套班子、培养一批人才、制定一套政策、整合一批资源”产业培育模式，落实“6+1+4”产业发展行动计划，提升轨道交通装备制造、有色金属深加工、化工、服饰加工贸易、医药食品、陶瓷等六个传统产业，培育物流产业，壮大汽车、新能源、航空航天和电子信息四大新兴产业，加快建成“四城两基地”，即电力机车城、汽车城、航空城、服饰城和国家有色金属深加工基地、新能源基地。壮大民营经济。出台鼓励民间投资的政策，支持引导民营企业发展，推进民营经济规模化、产业化、外向化。推进市属企业改革，鼓励民间资本介入。创造鼓励创业的良好环境，培育本土民营企业家。提升第三产业。从体制机制上研究芦淞服饰市场群提质改造的实施方案，全面提高市场群品位。实质性启动铜塘湾港区、国际物流园、芦淞服饰物流仓储配送中心，争取建设保税物流中心，大力发展现代物流业。扶持大型商业超市连锁经营，完善市场体系。加强房地产市场引导调控，促进房地产业健康发展。鼓励发展信息咨询、移动电子商务、服务外包、创意设计等新型服务业。

　　抓好园区攻坚。坚持突出特色、优势互补、错位发展，明确园区功能定位，优化园区布局，防止无序竞争。完善园区基础设施配套，增强园区承载功能，引导中小企业和民营企业向园区集中，招商引资产业项目向园区集聚。制定园区扶持政策和园区空间规划，重点建设轨道交通千亿产业园、风电产业园、汽车零部件加工园区、金山工业园二期、航空产业园、釉下五彩陶瓷创意产业园和服饰产业园。

　　着力扩大内需。加快项目建设，以项目带动投资，跟踪落实核电、中航发动机、山特维克、旗滨光伏玻璃、北大方正、黄海汽车等一批重大项目，抓好南车时代百亿工程、风电产业园、煤电一体化、北汽轿车等一批重点项目建设。主动对接央企和承接沿海地区产业转移，开展以商招商、上门招商、节会招商，积极引进战略投资者。扩大对外交流，积极参与上海“世博会”等经贸招商活动。落实国家促进消费的一系列政策，进一步做好家电、汽摩下乡工作。切实增强群众特别是低收入群体消费能力，不断优化消费环境。保持政策连续性，适应群众生活多样性、个性化的需要，引导消费结构升级，增强发展的内生动力。

　　促进旅游升温。发挥炎帝文化、工业文化、红色文化等特色文化优势，整合旅游资源，加快建设云峰湖国际度假区，综合开发炎帝陵、神农谷、云阳山、酒埠江、荷塘月色、大京等重点景区，完善旅游服务设施。引进知名旅游开发企业，组建旅游集团公司，加强旅游景点营销宣传，推动旅游市场持续升温。抢抓武广高铁通车机遇，加强区域合作互动，打响旅游品牌，提高旅游知名度，将株洲打造成粤湘鄂三省游客周末休闲的重要基地。

　　（三）突出城市提质，建设生态宜居家园

　　高水平规划城市。强化规划前瞻性、操作性和权威性，努力实现规划对经济社会发展、城市建设管理的全方位覆盖、全过程控制。严格按照“两型”社会要求，进一步搞好第七次城市总体规划修编及专项规划，完成天易示范区控制性详规和一批重点建设区域、重大建设项目的规划编制。加强城乡规划统筹，构筑中心城区、次中心城镇、小城镇协调发展的新型城镇体系。做好“十二五”规划编制工作。

　　高品位建设城市。按照“一体三极”战略构想，向北加快云龙示范区建设，向西加快武广新城建设，向南加快枫溪新城建设，全面拓展城市骨架。建成湘江五桥、湘江风光带河西城区段，拉通长江路，改造株醴路，加快东环北路等道路建设，启动建设神农大道、泰山路延伸段等一批城区道路，着力优化路网结构。新建和整合一批公交首末站场，完善智能化交通系统，抓好城区的污水处理、垃圾处理、停车场所等基础设施建设，切实完善城市功能。实施旧城区改造提质，重点推进城中村、棚户区改造，深入做好绿化、美化、亮化和数字化工作，尤其要着力提升城区的园林绿化水平，建设森林城市；完善功能分区和产业布局，城市四区各打造一条特色精品街，提升城市品位。坚持非均衡发展战略，以点带面，抓好县城和重点镇的提质扩容，扶持县城建设发展。抓好省直管县财政管理体制改革，拓宽县城开发建设投融资渠道，完善县城基础设施，不断提高县城首位度。

　　高标准管理城市。树立“大城管”理念，健全城市管理长效机制，拓宽考核范围，进一步完善运行市场化、管理网格化、作业精细化、考核标准化的城市管理体系。巩固创卫成果，深入整治城郊结合部、铁路进城沿线、农贸市场、小街小巷等薄弱部位环境卫生。深入推进城市创建工作，力争交通管理模范城市创建成功、环境保护模范城市指标基本达标，加快全国文明城市创建步伐。深入开展爱国卫生运动，认真开展卫生县城、卫生村镇、文明卫生单位创建活动。探索县城管理体制改革，增强县城发展活力。

　　（四）突出城乡统筹，夯实农村发展基础

　　统筹城乡产业发展。以增加农民收入为目标，加快农业结构战略性调整，加强县域经济建设。加快农业机械化步伐，实施新增粮食产能工程建设，开展粮食优质高产创建活动，确保粮食稳产增收。全面完成集体林权制度改革，绿化造林12.2万亩，其中“城边、路边、水边”造林4.45万亩。完善区域农产品布局规划，抓好106国道沿线优势农业和现代都市农业两大产业带建设。壮大炎陵白鹅、攸县麻鸭、茶陵烤烟和油茶等地方特色产业。以工业化的理念大力推进农业产业化，重点扶持唐人神、好棒美等产值过亿元的龙头加工企业。按照依法、有偿、自愿的原则，推进土地适度规模经营，鼓励发展各种专业合作社，建设一批无公害绿色食品及种苗花卉生产基地。建设区域性农产品加工中心、农产品流通中心，加快城乡农贸市场改造，健全农产品质量安全检测体系，强化食品安全监管，致力打造食品安全城市。

　　统筹城乡基础建设。加大农村基础设施建设投入，推进农村土地综合整治，推动城乡基础设施对接联网、互通共享。全面完成长株高速、醴潭高速株洲东互通至云田公路续建工程、醴潭高速芷线桥互通至渌口公路、省道S315龙下至攸县、网朱公路株洲县段建设，加快炎睦高速、炎陵分路口至县城高速、泉南高速茶陵县城至界化垅段、岳汝高速株洲段、国道106炎陵至槽里、省道S322炎陵至资兴段、省道S211株洲段建设，做好株潭快速南环线株洲段、省道S313渌口至湘潭谭家山公路建设的前期工作。提前实施“十二五”规划1000公里农村公路建设。加强农村地名标志设置工作。完成94座病险水库治理，抓好洮水水库建设扫尾和桐坝水电站建设，加快酒埠江、官庄、皮佳如、岩口大中型灌区的续建配套，开展洣水流域综合治理，全面推进小型农田水利设施建设。加强水文、气象防灾减灾设施建设。坚持基础先行、基地示范、产业带动、政府引导、市场运作原则，重点建设云田山庄、松西子、荷塘月色等新农村示范片，改善农村生产、生活环境。

　　统筹城乡公共服务。公共财政和新增财力重点向农村倾斜。加快发展农村医疗卫生、教育、社会保障等事业，完善农村困难群体生活保障体系，逐步实现城乡基本公共服务均等化。推动户籍制度改革，建立城乡统一的户口登记制度。做好失地农民的即征即保工作，维护失地农民的合法权益。加强技能培训，让更多农民成为有文化、懂技术、会经营的新型农民。扩大农村社区建设试点范围。

　　（五）突出以人为本，加快发展民生事业

　　发展社会事业。重视自主创新，坚持把自主创新作为转变发展方式和调整经济结构的中心环节，实现由“株洲制造”向“株洲创造”转变，积极发展战略性新兴产业。发挥企业创新的主导作用，建好国家轨道交通高分子材料中心，争取建设风电设备检验检测中心等一批研发中心和创新基地。以产学研结合为重点，完善科技创新体系，加快科技成果向现实生产力的转化。争取一批国家和省级科技项目，实施一批重点攻关项目，加大知识产权保护力度，促进专利产业化、产业链条化。利用城市提质的机遇，完善人才激励机制，引进一批高端人才创新创业。优先发展教育事业，深入推广“人文智育、阳光体育、快乐德育”教育理念。加快职教城建设进度，努力将株洲打造成“中部地区职业教育创新之都”。继续实施“阳光体育”工程，新建8个塑胶运动场。设立“安健教育奖”，营造全社会尊师重教的氛围。提质改造市博物馆和档案馆，启动炎帝神农艺术中心建设，继续推进乡镇综合文化站和农家书屋建设。抓好河东体育场改造，广泛开展全民健身运动，提高竞技体育水平。着力推进公立医院改革、医药卫生体制改革，以重大疾病和职业病预防为重点，完善公共卫生体系。整合医疗卫生资源，组建市中心医院、三三一医院和恺德医疗集团,启动二医院门诊大楼建设。规范新型农村合作医疗门诊统筹试点，提高新型农村合作医疗实效。搞好第六次全国人口普查，加强流动人口计划生育服务与管理，强化计生利益导向机制，统筹解决人口问题。切实维护残疾人和妇女儿童权益，大力发展社会福利、慈善、法律援助等社会公益事业。全面加强民族宗教、外事侨务、对台、新闻出版、地方志、档案等各项工作。

　　完善保障体系。继续实施积极的就业政策，广泛开展职业技能培训和创业培训，加大公益性就业岗位开发力度，全力解决困难群体就业问题。积极推进创业带动就业，努力使株洲成为要素集聚、功能配套的区域性创业中心，打造创业型城市。抓好社会保险扩面征缴工作，扎实做好新型农村养老保险工作和医疗保险的城乡统筹工作，实现城镇从业人员社会保险全覆盖，逐步健全统筹城乡的社会保险制度。建立临时生活困难救助制度，完善社会救助体系，增强社会救助能力。

　　维护社会稳定。强化信访工作责任制，加强和改进信访工作。实施“科技创安”、“社区创安”工程，严厉打击各种刑事犯罪活动，构建“平安株洲”。强化劳动保障监察执法，切实维护劳动者合法权益。加强价格调控，保持物价基本稳定。完善突发事件应急管理机制，健全新闻发布制度，提高处置突发事件和应对公共安全危机的快速反应能力。进一步抓好安全生产、森林防火工作，防止重特大安全生产事故发生。深入开展拥军优属、拥政爱民和军民共建活动，争创“全国双拥模范城”五连冠。加强全民国防教育，做好人民防空和民兵预备役工作。

　　因势利导重在执行纵深推进政府建设

　　提升素质，建设现代政府。紧扣现代政府“经济调节、市场监督、社会管理和公共服务”的职能目标，学习和掌握经济管理、金融管理以及资本运作等知识，拓宽视野，提升境界，增长才能，打造学习型政府。创新政府管理方式，立足国际化视野、市场化理念、前瞻性眼光，不断提高制度创新能力、项目策划能力和建设推动能力，打造建设型政府，让政府真正成为公共产品的提供者、优良环境的创造者、人民权利的维护者。

　　依法行政，建设法治政府。自觉接受市人大及其常委会的监督、市政协的民主监督、新闻舆论和社会公众监督，不断改进政府工作。认真办理人大代表建议、议案和政协委员提案，坚决做到件件有答复，事事有回音。畅通群众进言监督渠道，保障人民知情权、参与权、表达权和监督权。依法开展审计监督，突出抓好同级财政预算、政府投资项目和事关民生的专项资金的审计。规范行政自由裁量权，加强行政执法监督，提高依法行政能力。

　　强化执行，建设效能政府。严格执行科学民主的决策机制、精简效能的执行机制、奖惩分明的激励机制和权责统一的问责机制，培育执行文化。继续开展规范行政执法行为评议，完善督查工作机制，充分发挥“12345”市长热线作用，实现政务督查、行政监察、目标管理、民意传递和舆论监督联动。强化绩效考核，简化审批程序，优化工作流程，加快电子政务建设，提高服务效能。

　　规范管理，建设廉洁政府。严格执行廉洁从政各项规定，深入推进反腐倡廉工作。倡导程序简化、措施简便、材料简练、会议简短、接待简省、为人简单、生活简朴、下乡简从的“八简”作风，力戒铺张浪费，节约行政开支，降低行政成本。加快推进预防腐败体系建设，深化招投标体制改革，确保权力在阳光下运行。

　　各位代表！事业凝聚人心，信心昭示力量，行动铸就辉煌！站在新的起点，攀登新的高峰，我们信心百倍，坚定执着。让我们在市委的坚强领导下，同心同德，扎实工作，为实现“保二争一、科学跨越”战略目标而努力奋斗！
